# A dual pathways transfer model to account for changes in the radioactive caesium level in demersal and pelagic fish after the Fukushima Daï-ichi nuclear power plant accident

**DOI:** 10.1371/journal.pone.0172442

**Published:** 2017-03-01

**Authors:** Bruno Fiévet, Pascal Bailly-du-Bois, Philippe Laguionie, Mehdi Morillon, Mireille Arnaud, Pascal Cunin

**Affiliations:** 1 Laboratoire de Radioécologie de Cherbourg-Octeville, IRSN/PRP-ENV/SERIS, Cherbourg Octeville, France; 2 Laboratoire de recherche sur les transferts de radionucléides dans l'environnement, IRSN/PRP-ENV/SERIS, Saint-Paul Les Durance, France; University of South Carolina, UNITED STATES

## Abstract

The Fukushima Daï-ichi nuclear power plant (FDNPP) accident resulted in radioactive Cs being discharged into the local marine environment. While Cs bioaccumulates in biota and slowly depurates, the Cs concentrated in biota constitutes a source of Cs for animals feeding on each other. The marine biota therefore serves as a pool that recycles Cs, and this recycling process delays depuration in the fish feeding on this biota pool. Because the continental shelf is squeezed between the coast and very deep sea, the demersal marine species are confined to a narrow strip along the coast, close to the source of the radioactive input. Unlike demersal species, however, pelagic species are not restricted to the most contaminated area but instead spend some, if not most, of their time and feeding off-shore, far from the input source. We suggest that the feeding pathway for fish is a box whose size depends on their mobility, and that this feeding box is much larger and less contaminated (because of dilution through distance) for pelagic fish than for demersal fish. The aim of this paper is to test this hypothesis and to propose a simple operational model implementing two transfer routes: from seawater and from feeding. The model is then used to match the observational data in the aftermath of the FDNPP accident.

## Introduction

Since the Fukushima Daï-ichi nuclear power plant (FDNPP) accident in March 2011, radioactive discharges into the sea have involved two major phases. During the accident, when winds blew towards the sea, atmospheric releases (gas and aerosol particles) reached the surface of the Pacific Ocean [1–3]. Shortly afterwards, large volumes of highly contaminated water temporarily used to cool down the melted core inside the reactors during the emergency were released directly into the sea. This first phase began on 12 March and ended after a few weeks when the operator diverted the contaminated water into storage tanks for future decontamination. The main direct discharge into the sea occurred between 26 March and 8 April, 2011 [1]. A second discharge phase then began because of rain washout of the damaged FDNPP site as well as of the contaminated surrounding terrestrial catchment basin. However, the actual radioactive releases are incomparably smaller than the releases during the first acute phase.

Although the radioactive levels in seawater reflected these two main discharge phases, it was observed that demersal fish species (which live and feed on the continental shelf) exhibited slower depuration than pelagic fish species (which spend most of their time off-shore) [4–9]. At the same time, slow depuration was also observed in benthic invertebrates, which appeared to parallel the contamination level of the sediment [10]. In this paper, however, we shall focus on fish. Cs transfer to marine animals potentially involves three routes: via seawater, via sediment and via feeding. Two main hypotheses have been proposed to explain the difference between the depuration rates of demersal and pelagic fish. Slow depuration of the sediment compartment would influence the demersal species more than the pelagic species [10–13]. However, it is usually considered that transfer between sediment radioactive contamination and biota is only minor [14]. Another possibility is that different fish positions in the trophic web and feeding behaviours could result in different bioaccumulation and depuration profiles along the food chain [5–7, 9]. Regarding the food chain, it should be emphasised that the trophic web in the marine environment is generally considered to be unstructured and opportunistic [15, 16].

**Fig 1:**
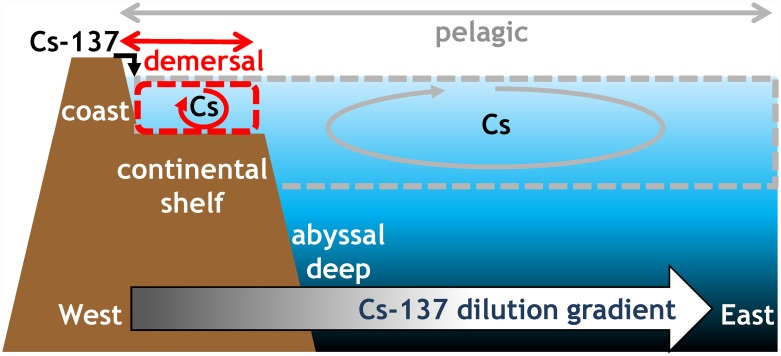
The marine environment, to the east of Japan, was schematised as two embedded boxes: A coastal box above the continental shelf (red dashed line box), where radiocaesium discharges from the FDNPP occur and demersal species are confined, bioaccumulate and locally recycle caesium within the food web, and a larger off-shore box (grey dashed-line box enclosing the coastal box) where the radiocaesium from the FDNPP is further diluted and pelagic species live far from the source but may be caught in the inner, coastal box.

Retaining the idea of an unstructured and opportunistic food chain, a simple hypothesis is explored here. While Cs bioaccumulates in biota and slowly depurates, the Cs concentrated in biota constitutes a source of Cs for animals feeding on each other. The marine biota therefore serves as a pool that recycles Cs, and this recycling process delays depuration in the fish feeding on this biota pool. Furthermore, this phenomenon is enhanced by the special conditions on the east coast of Japan. The continental shelf is squeezed between the coast and a subduction zone with very deep water. As a consequence, the benthic and demersal marine species are confined to a narrow strip along the east coast, close to the source of the radioactive input. However, unlike demersal species, pelagic species are not restricted to the most contaminated area but instead spend some, if not most, of their time and feeding off-shore, far from the source, where the contamination is diluted (Fig 1). As Cs accumulated in marine demersal species is recycled locally, near to the source of contamination, this local recycling slows the depuration of this biota Cs pool even further. In other words, the feeding pathway for fish constitutes a box whose size depends on their mobility, and this feeding box is much larger and less contaminated (because of dilution through distance) for pelagic fish than for demersal fish. The question concerns the relative contribution of both transfer pathways: via seawater and via feeding. We propose that these two transfer routes are enough to explain what is observed in demersal species as compared to pelagic species in Japan after the FDNPP accident. The aim of this paper is to test this hypothesis and to propose a simple operational model implementing the two transfer routes. We designed feeding boxes for demersal and pelagic fish and fit the model with observations off Japan after the FDNPP accident. Lastly, we attempted to qualify the model by statistically documenting the deviation between the calculated and observed values.

## Materials and methods

The marine environment to the east of Japan was schematised as two embedded boxes (Fig 1), consisting of a coastal box restricted to a narrow strip above the continental shelf, where (1) discharges from FDNPP occur (radiocaesium input), (2) demersal species are confined, bioaccumulate and locally recycle caesium within the food web, and (3) radiocaesium output occurs through hydrodynamic dilution away from the shore (outside the coastal box) and through pelagic species excursion between the coastal and off-shore areas. This box is contained within a much larger off-shore box in which (4) radiocaesium from the FDNPP is further diluted, resulting in lower levels than in the coastal box, and (5) pelagic species live away from the source but may be caught in the inner, coastal box.

A model has been designed to implement both transfer pathways, via seawater and via feeding, so that different kinetics can be considered for caesium dilution through hydrodynamics (with no barrier) and its recycling within the food compartment. This is confined to a coastal restricted area for demersal species but not for pelagic species.

### Model design

**Fig 2:**
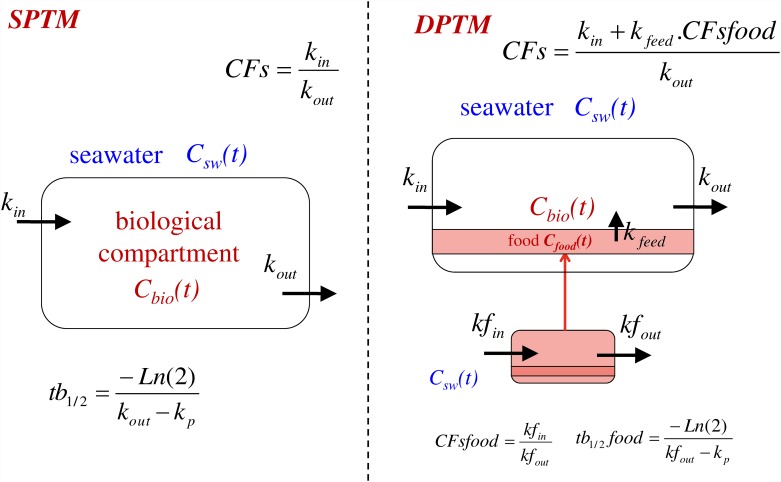
(Left) Single Pathway Transfer Model (SPTM) with two parameters k_in_ and k_out_ that account for the biological compartment input and output, respectively. (Right) Dual Pathways Transfer Model (DTPM) with five parameters: k_in_ and k_out_ accounting for the biological compartment input and output via the first pathway (seawater), kf_in_ and kf_out_ accounting for the food compartment input and output, respectively, and k_feed_ accounting for the contribution of the second pathway (feeding). CFs is the Concentration Factor (in steady state); tb_1/2_ is the biological half-life for the SPTM.

The Dual Pathways Transfer Model (DPTM), which implements the seawater and food pathways, is an extension of the Single Pathway Transfer Model (SPTM) previously described in [17]. Fig 2 compares the compartments, transfer routes and parameters implemented in these two models. It can be observed that the food compartment of the DPTM is described as an SPTM. It should be remembered that the SPTM is implemented for any radionuclide (RN) with the following equation [17]:

$$s_{i}=a.s_{i-1}+b.e_{i}$$ (1)

where *e*_*i*_ = [*RN*]_*seawater*_, *s*_*i*_ = [*RN*]_*biota*_ at time *t*_*i*_ and *t*_*i*_−*t*_*i*−1_ = *T* (the sampling period = model computing time step)

The key transfer parameters express from *a* and *b* as

 the Concentration Factor (in steady state) $CFs=\frac{b}{1-a}$
 and the biological half-life $tb_{1/2}=\frac{\text{ln}\left(2\right)}{k_{b}}$ with *k*_*b*_ = *k*_*out*_ − *k*_*p*_, (*k*_*p*_ the RN radioactive decay)
 and, since $a=exp^{\left[-k_{out}.T\right]},\;k_{b}=\frac{-\text{ln}\left(a\right)}{T}-k_{p}$

The DPTM is implemented using the same strategy as the SPTM, and the details for solving the differential equations and the mathematical background for the implementation of the solution are provided in the supplemental material (S1 Text and S1 Table). The final equations required to implement the DPTM are:

- The food compartment implemented as an SPTM by the equation
  $$f_{i}=a_{f}.f_{i-1}+b_{f}.e_{i},$$ (2)
  as described above (Eq 1), with *f*_*i*_ = [*RN*]_*food*_ at time *t*_*i*_.
- The Concentration Factor in food $CFsfood=\frac{b_{f}}{1-a_{f}}$ and the biological half-life of the food compartment $tb_{1/2}food=\frac{\text{ln}\left(2\right)}{k_{bf}},\;\;k_{bf}=\frac{-\text{ln}\left(a_{f}\right)}{T}-k_{p}$
- The consumer compartment is implemented as a DPTM using food (Eq 2) according to the equation
  $$s_{i}=a.s_{i-1}+b.e_{i}+c.f_{i}$$ (3)

To calculate [Cs-137] in the consumer *s*_*i*_, [Cs-137] in food *f*_*i*_ must be calculated (Eq 2) and introduced into the *s*_*i*_ equation (Eq 3) using [Cs-137] in seawater *e*_*i*_ and parameters *a*_*f*_, *b*_*f*_, *a*, *b* and *c*, which relate to CFsfood, tb_1/2_food, k_feed_, CFs and tb_1/2_ in accordance with S1 Table (*T* = 15 d in the present application).

Lastly, it was possible to use another signal such as sediment sed_i_ instead of seawater e_i_ as the input for the food compartment f_i_. This was done to test the potential contribution of sediment contamination to the levels in demersal fish near to the source (see Discussion section “Testing the DPTM model with other scenarios”).

#### The compartments implemented in the DPTM can be considered at different scales

The most intuitive scale considers one fish and its one prey. However, this would not match what was measured in the natural environment in the aftermath of the accident because the samples do not correspond to one fish and its one prey. The observation data account for samples from the entire fish population (not merely one fish), irrespective of species, age, behaviour and fish diet (not merely one prey), irrespective of the biological group (fish, crustaceans, molluscs, plankton, etc.). Moreover, the consumers and their food are supposed to be potentially mobile. In this context, the consumer compartment is “the fish population” and the food compartment is “all food of this fish population.” They are both assumed to move around inside the conceptual boxes as described in Fig 1. Consequently, the biological half-life of the food compartment (tb_1/2_food) accounts for Cs output from the entire diet of the fish population. This considered, it was then possible to connect the DPTM with the observation data in the marine environment after the FDNPP accident.

### Database of the marine environment following the FDNPP accident

DPTM parameters a_f_, b_f_, a, b and c could be determined by fitting the model to time series in seawater and biota as done previously with the SPTM model [17–19]. This was done by using the radioactive measurement data made available on the Internet by Japanese institutions after the accident.

#### Downloading from the Internet

Cs-137 concentration data in the marine environment were collected as read-only.pdf files and Excel^™^ worksheets from the websites of the Japanese Nuclear Regulation Authority (http://radioactivity.nsr.go.jp/en/list/205/list-1.html), Ministry of Agriculture, Forestry and Fisheries (http://www.jfa.maff.go.jp/e/inspection/index.html) and Tokyo Electric Power Company (TEPCO) (http://www.tepco.co.jp/en/nu/fukushima-np/f1/smp/index-e.html). Cs-137 concentrations values are expressed in Bq.L^-1^ in seawater, in Bq.Kg^-1^ (assumed dry) in sediment and in Bq.kg^-1^ wet in biota. In the very few cases where Cs-134 was given instead of Cs-137, either a typing error was suspected and discarded or Cs-137 was derived from the Cs-134 value on the basis of an assumed Cs-134/Cs-137 ratio of 1 on 11 March 2011, with respect to their differential radioactive decay.

For seawater and sediment, the geographical coordinates (longitude E, latitude N) of the sampling locations were provided. For biota, however, these coordinates were seldom documented although information was provided either on the prefecture of the landing port or on the town in front of which the data was obtained. On the basis of this loose information, biota data were geographically referenced according to the following arbitrary rules, with respect to the Japan’s east coast:

- Town or landing port: 3 km eastward;
- Prefecture: 50 km eastward at the median latitude;
- Region/Area (several prefectures): 50 km eastward at the median latitude.

It should be emphasised that for mobile species like fish, the capture location would only be indicative of the exposure level in seawater at the time they were caught. The seawater Cs-137 level they were exposed to prior to their capture remained unknown. Also, the seawater Cs-137 level to which mobile species were exposed to during the acute initial phase of the accident remained unknown, but aggregating data from the [2–30] Km area was a way to minimize the effects of these sources of uncertainty. However, the robustness of this method was poor and caution should be taken regarding the sampling location of biota data.

#### Dataset extraction

**Fig 3:**
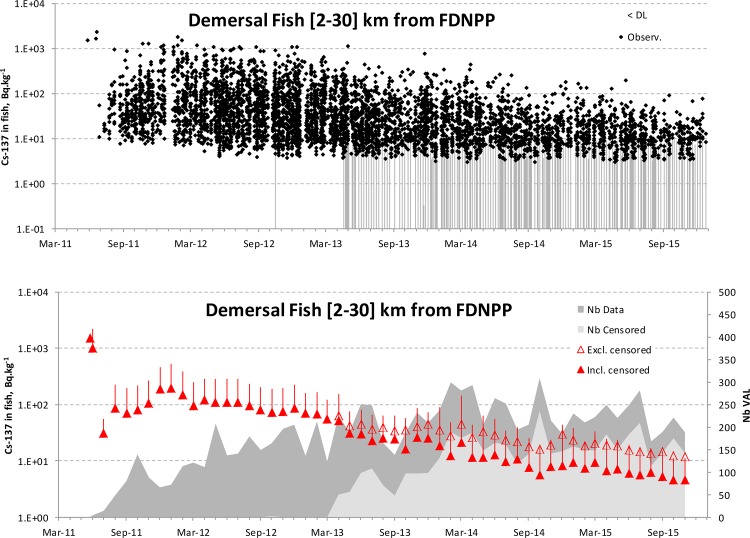
**Upper panel**: Raw data of Cs-137 values (left Y-axis in log scale) in demersal fish from the near field ([2-30] km from FDNPP); black dots: values above the LoD; vertical bars: censored values (<LoD). **Lower panel**: Open triangle: monthly averaged levels in fish computed without the censored values; solid triangle and vertical bars: monthly averages + SD computed with the censored values. Dark grey and light grey areas (right Y-axis): the total number of values and the number of censored values, respectively.

To demonstrate its relevancy, the DPTM was challenged to reproduce the Cs-137 contamination of fish after the FDNPP accident, including the difference between demersal and pelagic species. Datasets were extracted from the databases for that purpose in order to derive signals in seawater, sediment and biota groups for use as time series e_i_, s_i_. This included the following (see map in Fig 3 right panel):

*Datasets in the physical compartments (seawater*, *sediment)*:

- [Cs-137] in near-field seawater: data were selected on the basis of their sampling locations between 2 and 30 km from FDNPP (the FDNPP coordinates were set to longitude = +141.0348°E; latitude = +37.4249°N). This selection was hereafter referred to as “[2–30] km near-field”. These boundaries were chosen arbitrarily: the 2 km minimum discarded the immediate influence of the FDNPP harbour, where high concentrations occasionally occur, while the 30 km semicircular maximum arbitrarily delineated a near-field area that included a large number of values along with time in all three compartments (seawater, sediment, demersal and pelagic fish);
- [Cs-137] in sediment in the near field: the sediment dataset referred to as “[2–30] km near-field”;
- [Cs-137] in seawater off-shore: A rectangular box was arbitrarily defined between longitudes 141.6°E to 144.5°E and latitudes 35.3°N to 38.6°N. This selection was hereafter referred to as the "off-shore box". Although the NW corner is not far from the coast, the intention was to combine sampling locations far from the FDNPP as well as enough data in seawater and in pelagic fish.

*Datasets in biota (fish only)*:

- [Cs-137] in demersal fish in the near field: data were selected on the basis of the fish species and qualified as demersal or pelagic on the basis of the information collected from Fishbase (http://www.fishbase.org) as well as relevant publications by Japanese colleagues [4];
- [Cs-137] in pelagic fish in the near field: see above. Pelagic fish were supposed to stay and feed off-shore for most of their time but could be caught within 30 km of the coast. It should be noted that geo-referencing of fish sample locations was often based on the landing port, meaning that pelagic fish included in the near-field dataset might conceivably have been caught beyond the 30 km boundary;
- [Cs-137] in pelagic fish off-shore: see above regarding pelagic fish from the off-shore box, with similar inherent uncertainties.

The raw data used to test the DPTM are provided in spreadsheets as supporting information (S1 Data). It should be emphasised that these dataset extracts were not supposed to geographically account for the conceptual coastal and off-shore feeding boxes introduced in Fig 1 but were designed to provide sample data representing events in those boxes. Selected data in fish included both teleost and elasmobranch species. To our knowledge, the distinction between teleost and elasmobranch as regards Cs metabolism is unlikely to be enough documented to justify a separation, especially when both the seawater and feeding pathways are considered (not specifically osmoregulation). So far, marine radioecology didn't make the distinction between teleost and elasmobranch fish for the recommendation of Concentration Factor values ([20, 21]). The strategy was meant to aggregate enough data to build time series with evenly distributed information (over time and space) as far as possible. The results presented here account for teleost and elasmobranch fish species, together. Nevertheless, it is likely that slightly different results may be obtained if the number of data would allow considering teleost and elasmobranch separately and if the knowledge of the exposure level would be more accurate.

### Building time series for the model and dealing with non-detection

The values from the seawater and sediment datasets were aggregated to produce the necessary signals for input into the model. Data were averaged on a monthly basis to build a periodic time series, and linear interpolation was used to build an input signal with a sample period T of 15 d. In order to fit the model to the output signal, the values in fish datasets were also aggregated as monthly averages. The datasets potentially included values reported as <LoD (Limit of Detection) referred to as “censored values" or "non-detects” [22]. When building the signals for the model, non-detects were dealt with as previously described [23]. A 80% cut-off threshold [22] was used to discard monthly sub-datasets including too many censored values. Data were processed using the R language and programming environment for statistical data analysis [24], along with the add-on NADA package [25–27].

### Determining the DPTM parameters

The DPTM parameters a_f_, b_f_, a, b and c, from which CFsfood, tb_1/2_food, k_feed_, CFs and tb_1/2_ can be derived could be determined using the least squares method (or any other fitting criteria). However, using a mathematical fit criterion of any type to derive five parameters from such loose information would not ensure consistent and reliable results. Data aggregation based on distance ([2–30] km from the FDNPP) or a large off-shore box, data aggregation from many species and the weakness of the fish localisation information all result in very large uncertainties regarding the signals used both as the model input and as fish data to match with the model output. In other words, a similar best fit can be computed with many sets of all five parameter values. Alternatively, it was possible to integrate information from the literature and so set constraints and reduce the number of unknowns. This was the case for the CFs value in marine fish, which is already well documented for Cs-137, and there was no reason to challenge the recommended value of 100 [20] although this comes from a range of values reported in the literature. Accordingly, the CFs value was set to 100, and the CFsfood value was also set to 100 so the model computes Cs-137 levels in predator fish feeding on fish preys. It should be noted that other types of preys may be considered, consisting of recommended values in other marine biota groups for CFsfood, such as 50 or 60 for crustaceans or molluscs, respectively [20]. Three transfer parameters remained to be determined: tb_1/2_, k_feed_ and tb_1/2_food. Although it provided an initial estimation, using a residual minimisation technique (absolute or relative) was not considered the most appropriate method. These three transfer parameters were instead fitted by eye to match different datasets (near-field/off-shore; demersal/pelagic fish) on the basis of the considerations explained in the Discussion section. Moreover, the number of significant digits of these transfer parameters was voluntarily set to the minimum (1 or 2) because higher-resolution values would not make sense with such loose environmental data, as stated above. Nevertheless, the model’s sensitivity to the parameter values was specifically addressed in the Discussion section (“DTPM sensitivity to transfer parameter values”) in order to evaluate the reliability that can reasonably be considered regarding these parameter values.

### Qualification of model performance

To qualify DPTM performance, the residuals between the model and the observations were statistically analysed. Two parameters (Rc and R) were calculated for each available individual observation data (Obs) and its corresponding value calculated by the DPTM (Mod), as previously described [28]. The Mod value corresponding to each considered Obs value was taken as the model value computed for the previous (or equal) time step of the model, on the T = 15 d pace basis.

*if Mod* < *Obs then $Rc=1-\frac{Obs}{Mod}$ else $Rc=\;\frac{Mod}{Obs}-1$*

The Rc value equals 0 when Mod and Obs exactly match, otherwise Mod and Obs are compared using their ratio showing a relative residual (an absolute residual would have weight depending on the level). The range is linear and symmetrical with negative values and positive values corresponding to an underestimate and overestimate of the model, respectively. For example, a two-fold overestimate by the model yields Rc = +1 while a two-fold underestimation yields Rc = -1. A histogram can then be plotted to characterise the model’s ability to yield symmetrical Rc values centered on 0.

To complete the statistical analysis of the relative residual, R is calculated as

R = Max(Obs/Mod; Mod/Obs) $R=Max\left(\frac{Obs}{Mod};\frac{Mod}{Obs}\right)$

R equals 1 when Mod and Obs exactly match. A histogram of R values characterises the factor by which the model and the observations mismatch if the model under- or over-estimates the value. A cumulative percentage of the distribution characterises the probability of the model being wrong by less than a factor n. For example, it provides the probability of being wrong by less than a factor 2 or by less than one order of magnitude (n = 10). Minimising the sum of the R value was also a potential fitting criterion for determining the model parameters.

## Results

### Time series datasets used to test the model

Fig 3 shows an example of a dataset extraction with the raw data and the monthly averages excluding and including the censored values (<LoD). Taking into account the censored values clearly yielded a different signal in demersal fish from the near field as of 2013. This signal was preferred because it was considered to be more representative of realistic mean levels. S1, S2 and S3 Figs display the datasets extracted from the database in detail and the corresponding derived input signals in seawater (S1 Fig: near-field; S2 Fig: off-shore) and sediment (S3 Fig: near-field). S4 Fig displays the datasets extracted for pelagic fish from the near field and the corresponding monthly averages, dealing with the censored values.

**Fig 4:**
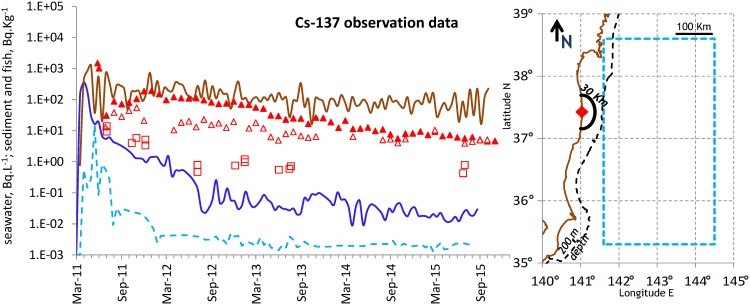
**Left panel**: Signals in near-field seawater (solid blue line), off-shore box seawater (dashed blue line) and near-field sediment (solid brown line) derived from monthly averages and used as the input for the DPTM. Monthly averages in near-field demersal (solid triangles) and pelagic (open triangles) fish and individual values in off-shore box pelagic fish (open squares). **Right panel**: Map in (longitude N, latitude E) coordinates, displaying the east coast of Japan with FDNPP (red diamond), isobaths 200 m (dashed black line), the coastal near-field semicircle box (solid black line) and the rectangle off-shore box (dashed light blue line) delineating the areas for selecting seawater and fish data.

Fig 4 summarises the signals derived from seawater and sediment datasets used as the input for the DPTM and the monthly averages or individual values derived from the fish datasets. The right panel displays a map with the near-field and off-shore areas designed to select the datasets.

### Testing and qualifying the DPTM with demersal fish in the near-field box

**Fig 5:**
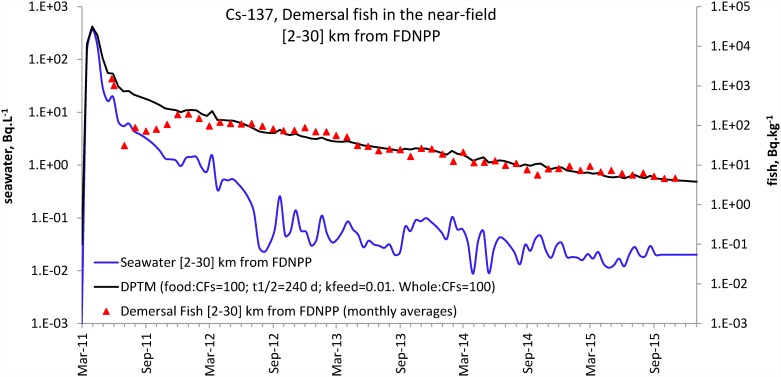
DPTM (black solid line) for demersal fish in the near field (solid triangle) using the near-field seawater signal (blue line) as the input. CFsfood and CFs were set to 100. Transfer parameters were tb_1/2_ = 5 d; k_feed_ = 0.01 d^-1^; tb_1/2_food = 240 d.

**Fig 6:**
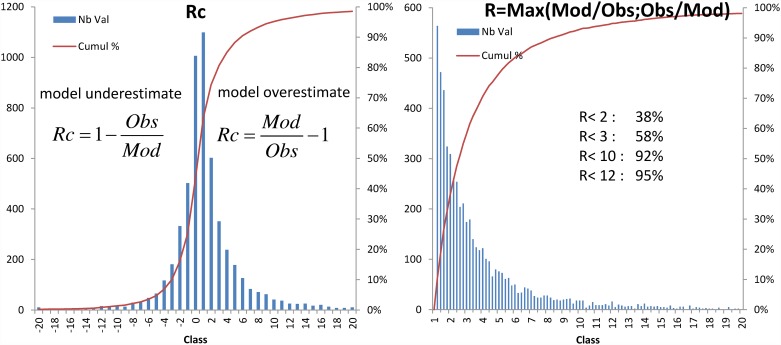
Histograms and cumulative percentages of the distributions of Rc and R values, showing the performance of the DPTM on demersal near-field fish presented in Fig 5.

The DPTM was tested with the constraints CFsfood and CFs = 100 with the aim of modelling fish prey consumption by demersal predator fish. Computing the best fit by minimising R (R> = 1) yielded an initial estimate of the parameters tb_1/2_ = 2.3 d, tb_1/2_food = 286 d and k_feed_ = 0.016 d^-1^. Fig 5 displays the DPTM response results with a half-life for the water pathway (tb_1/2_) of 5 d, a half-life for the food pathway (tb_1/2_food) of 240 d (8 months) and a k_feed_ value of 0.01 d^-1^. The choice of those parameter values, which were fitted by eye and differ from the optimum fit estimates, is explained in the method section and argued further in the discussion section. In order to assess the performance of the model, the histograms and cumulative percentages of the distributions of Rc and R values (based on individual data) are presented in Fig 6. The Rc histogram was symmetrically distributed and centred a little above Rc = 0, with a cumulative percentage value Rc_50%_ of 0.33. This meant that with this set of parameters, the DPTM slightly overestimated by +33% on average, which was conservative as regards radioprotection. The R cumulative percentage showed that a mismatch of less than a factor of 2 was obtained in 38% of the data and the order of magnitude was correct in 92% of the data.

### Testing the DPTM with pelagic fish from the off-shore box

**Fig 7:**
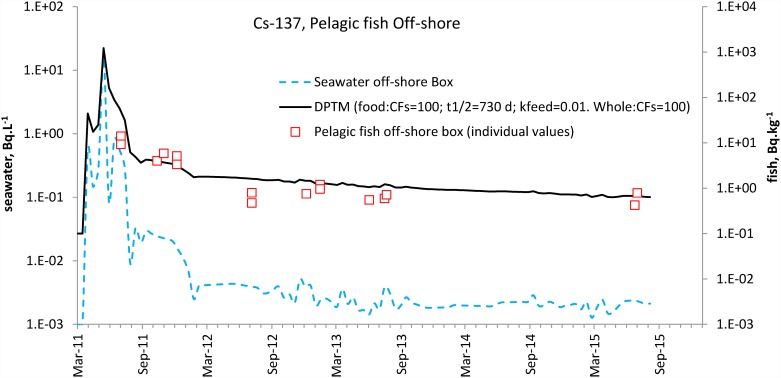
DPTM (black solid line) for pelagic fish in the off-shore box (open square) using the off-shore seawater signal (blue dashed line) as the input. CFsfood and CFs were set to 100. Transfer parameters were tb_1/2_ = 5 d; k_feed_ = 0.01 d^-1^; tb_1/2_food = 730 d.

Fig 7 shows the DPTM using the monthly averaged data for the off-shore box seawater as the input signal and the same transfer parameters as for demersal fish from the near field, except for tb_1/2_food for the food pathway: CFsfood and CFs = 100; tb_1/2_ of the water pathway = 5 d, tb_1/2_food of the food pathway = 730 d (2 years) and k_feed_ = 0.01 d^-1^. It should be noted that data in pelagic fish from the off-shore box were individual values and not monthly averages.

### Testing the DPTM with pelagic fish from the near field

**Fig 8:**
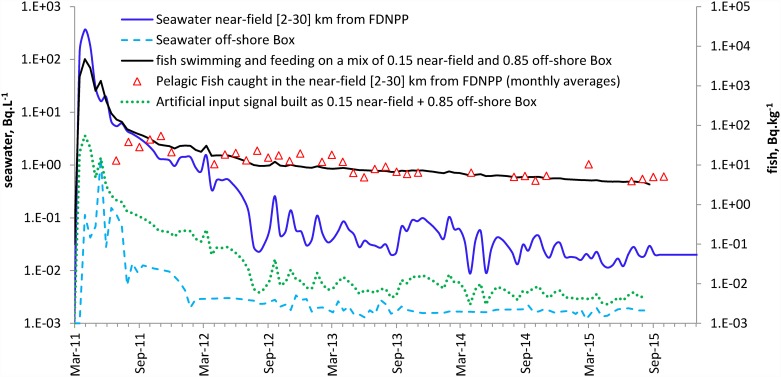
DPTM fit (black solid line) to pelagic fish caught in the near field (open triangles) using an artificial combination of the near-field signal (blue solid line) and the off-shore signal (blue dashed line) in seawater as the input (green dotted line). CFsfood and CFs were kept to 100. Transfer parameters were tb_1/2_ = 5 d; k_feed_ = 0.01 d^-1^; tb_1/2_food = 730 d.

Another challenging question arising from observations following the FDNPP accident was the difference in depuration for pelagic fish and for demersal fish. The DPTM proved to be effective in computing the levels in demersal fish from the levels in seawater in the near field and the levels in pelagic fish from the levels in seawater in the off-shore box. The model, therefore, reliably predicted the levels in fish on the basis of their local levels in seawater. Our primary hypothesis was that, unlike demersal species, pelagic species caught in the near field are not restricted to the most contaminated area but instead spend some, if not most, of their time and feeding off-shore. The input signal (in seawater) in the DPTM for pelagic fish caught in the near field should therefore be a mix of the seawater levels in the near field and off-shore. It was not possible to design a box that extends from the near field to off-shore and which contains an even data distribution (in terms of time and space) from the seawater database. Furthermore, it was not possible for the input signal to reflect the portion of time spent in the near field and off-shore by pelagic fish. As a result, an artificial input signal was built by combining fractions of the near-field and off-shore box input signals. For example, an equal mix can be computed as 0.5 near field + 0.5 off-shore box, which means that fish spent half their time in the near field and the other half in the off-shore box. We are well aware of the artificial nature of this computed input signal, but it was merely designed to challenge the DPTM’s ability to fit the data of pelagic fish caught in the near field by reducing the input signal (the exposure level of seawater and food). Furthermore, it was what could be expected if pelagic fish caught in the near field divided their time between the inshore and off-shore areas. Fig 8 shows the DPTM using 0.15 near-field + 0.85 off-shore box seawater signal values as the input. The same transfer parameters as for pelagic fish in the off-shore box were retained to match the model output with the monthly averages in pelagic fish caught in the near field.

## Discussion

### DTPM sensitivity to transfer parameter values

The DTPM best fit of the demersal fish near-field dataset was computed by minimising the sum of the relative residual R (R > = 1) as an initial estimate of the parameters. The constraints were set for CFs and CFsfood = 100, and the best fit was obtained with tb_1/2_ = 2.3 d, tb_1/2_food = 286 d and k_feed_ = 0.016 d^-1^. This best fit was driven by the sharp decrease in fish in 2011, which yielded the low value tb_1/2_ = 2.3 d. This low value of tb_1/2_ is then balanced by the value of k_feed_ (0.016 d^-1^) to match the levels in fish after 2011. During the few weeks after the accident, very high seawater levels of Cs-137 were observed in the near field (Fig 5 and S1 Fig). Though we lack data in fish just after the accident, the contamination level of demersal fish observed in late 2011 (Fig 4) meant that the very high levels in seawater were not reflected in biota for a long time. This was possible only if tb_1/2_ of the seawater pathway was short, thereby explaining the low value of tb_1/2_ (2.3 d) proposed by the best fit. Values reported in the literature for tb_1/2_ of the seawater pathway for Cs-137 in fish (excluding feeding) are scarce and vary widely [4–7, 9, 12, 29]. The notable uncertainties in late 2011 regarding both signals in seawater (many censored values) and fish (few data) and the loose geographical references demand caution. Raising the value of tb_1/2_ to 10 d yielded an overestimate of the level in fish by the model in 2011 (not shown). For these reasons, fitting by eye was preferred and a value of tb_1/2_ was arbitrarily set to 5 d in a realistic range [1–10]. The value of k_feed_ was closely related to this chosen value because it gives the general level of the model output in fish after 2011. In fact, once tb_1/2_ was set to 5 d, a better fit was obtained with k_feed_ = 0.01 d^-1^, which was close to the best-fit value of 0.016 d^-1^. The value of tb_1/2_food gives the general slope of the decrease in the fish level over the four-year period. The data’s time span yielded a fairly robust value of 240 d (8 months) which fitted better than 289 d once tb_1/2_ and k_feed_ were set to 5 d and 0.01 d^-1^, respectively. The value of k_feed_ (0.01 d^-1^) can be viewed as an incorporation rate of Cs in fish through feeding that could be determined in laboratory experiments using radiocaesium-labelled food. To the best of our knowledge, the literature is scarce on this topic but our value of 0.01 d^-1^ exactly matched the accumulation rate of Cs-137 via food to the whole body in the plaice and the brown trout (in seawater) reported by [30].

In the case of demersal fish from the near field, despite the sources of uncertainty (space and species aggregation), the model’s visual overlap and the monthly averages for the years spanned since 2012 were indicative of a good match. The reliability of the model was also demonstrated by statistical analysis of the residual between the model calculations and the individual observations, which yielded a less than two-fold mismatch in 38% of cases. A transient mismatch between the model and the observations was apparent in 2011 (Fig 5). There again, there were few values in fish in 2011, and the derived monthly averages were likely to poorly represent the actual levels (Fig 4). Furthermore, most of the censored values in the seawater dataset were between summer 2011 and spring 2012 (S1 Fig) and could not be taken into account since they were more than 80%. The input signal was thus derived solely from values above the LoD, which probably overestimated the actual level in seawater and, consequently, the model’s computed level in fish. Obviously the lack of accurate knowledge of what happened in seawater and local fish near the power plant in the very first weeks of the accident was detrimental.

### Difference in demersal and pelagic fish depuration

The visual overlap of the model and the individual data in pelagic fish caught off-shore (Fig 7) for the years under consideration was satisfactory. This application of the DPTM to a different area supported the model’s validity and suggested that the values of tb_1/2_ = 5 d set for the water pathway and k_feed_ = 0.01 d^-1^ seemed appropriate. Merely swapping the input signal (concentration in seawater) in the DPTM from the near field to the off-shore box provided a good estimate of the concentrations in pelagic fish from the off-shore box. Tripling the half-life of the food pathway (tb_1/2_food) to 730 d (two years) flattened the DPTM response in fish, which provided a better visual match with the few observations up to 2015. The choice of the 730 d value was justified by the need to set a long tb_1/2_food value to fit the slope and the looseness of the environment data dictating a limited number of significant digits, and so it was arbitrarily set to two years.

The model implements two routes: the water pathway and the trophic pathway. The water pathway is driven by the Cs-137 concentration in seawater and the dilution through hydrodynamics occurring in the water mass off-shore from Japan. Even with the stratification of the Northwest Pacific Ocean, which primarily offered the top water layer for dilution, the volume is very large. There is also no particular reason why the physiological seawater Cs transfer process should greatly vary in fish whatever their life mode (demersal or pelagic), and so the same value was used throughout this study (tb_1/2_ = 5 d). The relative contributions of seawater and feeding pathways were also not expected to differ greatly for demersal and pelagic fish. This contribution is implemented through k_feed_, and this parameter value of 0.01 d^-1^ was used in all test case implementations of the DPTM. However, the main factor that differed between the demersal and the pelagic fish species was the exposure level through seawater and feeding. The seawater level used as the input signal for the model turned out to be crucial in providing lower concentrations in pelagic fish than in demersal fish, regardless of whether they were caught in the near field or in the off-shore box. Lastly, the half-life of Cs in the food box also proved to be crucial. In the near field, recycling of Cs in a small box above the continental shelf was implemented with a tb_1/2_food of 240 d (eight months). For pelagic fish, whether they were caught in the near field or off-shore, Cs recycling was implemented with tripled half-life (two years). This agrees with the fact that pelagic food represents a much larger food box than the one confined to the narrow strip along the coast, in which demersal biota feed (i.e., the larger the food box, the longer it takes to depurate). The transfer parameters used for pelagic fish were the same both in the near field and off-shore. The only difference was the input signal in seawater, which was estimated on the basis of measurements in the off-shore box and artificially built in the near field to account for the transient time spent in the near field where they were caught.

### Testing another choice for the database extract areas

The areas chosen to represent what occurred in the conceptual feeding coastal and off-shore boxes were empirically selected but designed to contain enough data in the vectors (seawater and sediment) and in fish with an equal space and time distribution over the whole period. However, we wondered whether a different choice would have yielded different values for the transfer parameters. This issue was explored by designing another coastal area as a narrow strip along the east coast of Japan between latitudes 35.7 N and 38.3 N and delineated by isobaths 200 m. The data were extracted and processed in this coastal strip in the same way as in the 30 km in the FDNPP semicircular coastal area. The results presented in S5 Fig showed that the DPTM with the same transfer parameters (CFsfood and CFs = 100; tb_1/2_ = 5 d; k_feed_ = 0.01 d^-1^; tb_1/2_food = 240 d) also fitted very well with this alternative dataset.

### Testing the DPTM with other scenarios

#### Influence of the sediment compartment

Slow depuration was observed in benthic invertebrates, which seemed to parallel the contamination level of sediment [10]. Slow depuration was also observed in sedentary demersal fish in the near field [4, 8] and the hypothesis of an influence by sediment contamination levels naturally emerged [13], although it was reported that transfer between sediment radioactive Cs contamination and biota is minor [14]. However, we used the DPTM to test the potential effect of a transfer route influenced by sediment. To implement this process, the near-field sediment signal (S3 Fig and Fig 3) was used instead of seawater to compute the food pathway as *f*_*i*_ = *a*_*f*_ · *f*_*i*−1_ + *b*_*f*_ · *sed*_*i*_. This would mean that the demersal fish prey signal depends entirely on sediment, which would obviously be an extreme approach. When doing so, the model provided a poor match for the signal in demersal fish because the model’s output failed to reproduce the depuration slope for any parameter values in fish (**not shown**). The explanation lies in the general trend of the signal in sediment, which was stable over the period but decreased in fish. This trial application did not rule out the sediment compartment’s possible influence upon demersal fish in the near field, but rather suggested that it was probably not the major process governing Cs transfer to fish after the FDNPP accident.

#### Sources of food other than fish prey

It was tempting to challenge the DPTM with the scenario of predator fish feeding on food other than fish. As described in the methods section, this was easily tested by using another value of CFsfood recommended in the literature, such as 50 or 60 for crustaceans or molluscs, respectively (IAEA TRS422, 2004). When setting CFsfood = 50 instead of 100, the model output was exactly halved. As a result, in order to fit with the signal in the consumer fish, it was necessary to double k_feed_. There was a simple inverse relationship between CFsfood and k_feed_: changing CFsfood by factor X and k_feed_ by 1/X yielded the same output signal by the model. This was not surprising because of the equation expressing CFs and its dependence on CFsfood and k_feed_ (S1 Table). This illustrates the DPTM’s ability to handle other sources of food.

#### Testing the DPTM model designed in Japan with fish data from the English Channel

A final test was performed to challenge the DPTM in a completely different geographical context. Because the Areva NC La Hague nuclear reprocessing plant (France, Normandy) releases controlled liquid discharges in normal operation, the operator monitors Cs-137 levels in fish from the English Channel. At the same time, a hydrodynamic model is also available to compute the dispersion of radionuclides in the Channel on the basis of discharge data. Thanks to this accurate knowledge of discharges and the thousands of measurements of radioactivity in seawater taken by IRSN over the past decades, this model has been fine-tuned, well-validated and predicts the concentrations in seawater with an outstanding reliability of less than a factor of 3.6 between Mod and Obs where p = 95% [28]. The oceanographic characteristics of the English Channel differ from Japan, as this entire Channel is entirely located on the continental shelf between the coasts of France and the UK. Sea fish in the Channel can be characterised as demersal or coastal (but this does not exclude the presence of pelagic species), as in Japan in the near field of the FDNPP. In this context, it was tempting to use the DPTM and its transfer parameters obtained in Japan. The input signal for the DPTM was not based on measurements in seawater but on the calculations using the hydrodynamic model [28]. The transfer parameters were the same as for demersal fish in the near field of the FDNPP (CFsfood and CFs = 100; tb_1/2_ for the water pathway = 5 d, tb_1/2_food for the food pathway = 240 d and k_feed_ = 0.01 d^-1^). This trial application with the input signal produced by the hydrodynamic model, the individual monthly measurement values in demersal fish from 1986 to 2004 and the results of the DPTM are presented as supporting information (S6 Fig). As with Japan, the qualification analysis yielded a mismatch between Mod and Obs of a factor of less than 2 in 79% of the data and less than 3.3 in 95% of the data. This trial, which has spanned almost two decades, strongly supported the validation of the DPTM initially implemented in Japan as a robust tool for modelling Cs transfer to fish in a marine environment.

## Conclusion

This paper has demonstrated that the Dual Pathways Transfer Model is likely to reproduce the main processes governing the transfer of Cs to fish in the marine environment following the Fukushima nuclear power plant accident. It has proved itself reliable both in an accidental context (Fukushima) and in normal operation (English Channel). The model has also successfully implemented the hypothesis proposed in the introduction to explain the difference between the depuration rates of pelagic and demersal fish species, simply based on the transient time spent in the near field by pelagic fish. The model’s output depends on the input signal and the values of five transfer parameters: CFs, tb_1/2_ for the seawater pathway, CFsfood, k_feed_ and tb_1/2_food for the trophic pathway. Those parameters can be considered as three groups:

1. CFs and CFsfood account for the Cs-137 steady-state Concentration Factor values (100 in fish) that can be obtained from the literature with confidence because they are well documented.
2. tb_1/2_ and k_feed_ dictate the relative contribution of the seawater and trophic routes. These are closely related, as changing the value of tb_1/2_ involved changing the value of k_feed_ to maintain a good match with the observation data when fitting the model. This study produced consistent results if the same (tb_1/2_; k_feed_) pair was maintained for both demersal and pelagic fish. This makes sense, as these two parameters are closely related to the physiology of fish regarding Cs transfer and there is no reason why this should depend on the fish’s location (coastal or off-shore) when data from many different fish species are aggregated. Furthermore, the fact that the same (tb_1/2_; k_feed_) pair could be used successfully in all study cases to match the model and the observations supports the choice of the values tb_1/2_ = 5 d and k_feed_ = 0.01 d^-1^. Care should be taken if looking at individual species, however.
3. tb_1/2_food was found to be a parameter discriminating between demersal (coastal) and pelagic fish datasets, provided the appropriate input signal is used. We would like to emphasise that the longer tb_1/2_food for pelagic fish (two years) and the shorter one for demersal fish (eight months) describe the Cs turnover in food boxes and not in individual fish. This does not mean that pelagic fish species have a slower Cs biological half-life than demersal fish species, but rather that the much larger pelagic food box takes longer to empty than the small demersal food box contained above the continental shelf. This does not contradict the observation that the apparent depuration of pelagic fish is faster than that of demersal fish in the near field. In fact, the very factor driving the apparent depuration of demersal and pelagic fish caught in the near field is the input signal, which is the general exposure level (via both the water and food) and is certainly not the same for both types of fish.

The DPTM could also be used to test the possible influence of the contamination remaining in sediment on the continental shelf on Cs-137 kinetics in fish after the FDNPP accident. Although this component should not be ignored, its contribution to the slower depuration observed in demersal fish is probably minor.

## Supporting information

S1 Data Microsoft EXCEL^™^ file with spreadsheets of all Cs-137 concentration raw data in seawater, sediment and fish used to test the DPTM as well as fish species and their qualification as demersal or pelagic. (XLSX)

S1 Fig Database extraction for the seawater (near field). (PDF)

S2 Fig Database extraction for the seawater (Off-shore). (PDF)

S3 Fig Database extraction for the sediment (near field). (PDF)

S4 Fig Database extraction for pelagic fish caught in the near field ([2–30] km from the FDNPP). (PDF)

S5 Fig DPTM testing with an alternative dataset for the near-field area delineated between latitudes 35.7 N and 38.3 N and 200m isobaths. (PDF)

S6 Fig Testing the DPTM in the English Channel for demersal fish with the same parameters as in Japan. (PDF)

S1 Table Equations relating to the transfer parameters and the model values of a, b, af, bf and c. (PDF)

S1 Text Mathematical background for the DPTM including the differential equations and the implementation of their solutions. (PDF)
