# Correction: A dual pathways transfer model to account for changes in the radioactive caesium level in demersal and pelagic fish after the Fukushima Daï-ichi nuclear power plant accident

**DOI:** 10.1371/journal.pone.0175614

**Published:** 2017-04-06

**Authors:** Bruno Fiévet, Pascal Bailly du Bois, Philippe Laguionie, Mehdi Morillon, Mireille Arnaud, Pascal Cunin

The second author’s name appears incorrectly in the published article. The correct name is: Pascal Bailly du Bois. The correct citation is: Fiévet B, Bailly du Bois P, Laguionie P, Morillon M, Arnaud M, Cunin P (2017) A dual pathways transfer model to account for changes in the radioactive caesium level in demersal and pelagic fish after the Fukushima Daï-ichi nuclear power plant accident. PLoS ONE 12(3): e0172442. doi:10.1371/journal.pone.0172442.
