# Supplementary material for: A dual pathways transfer model to account for changes in the radioactive caesium level in demersal and pelagic fish after the Fukushima Daï-ichi nuclear power plant accident
Source: PLoS One. 2017 Mar 1;12(3):e0172442. doi: 10.1371/journal.pone.0172442 (PMC5383001; doi:10.1371/journal.pone.0172442)
Supplement: S3 Fig — (PDF) [file pone.0172442.s004.pdf]

S3 Fig

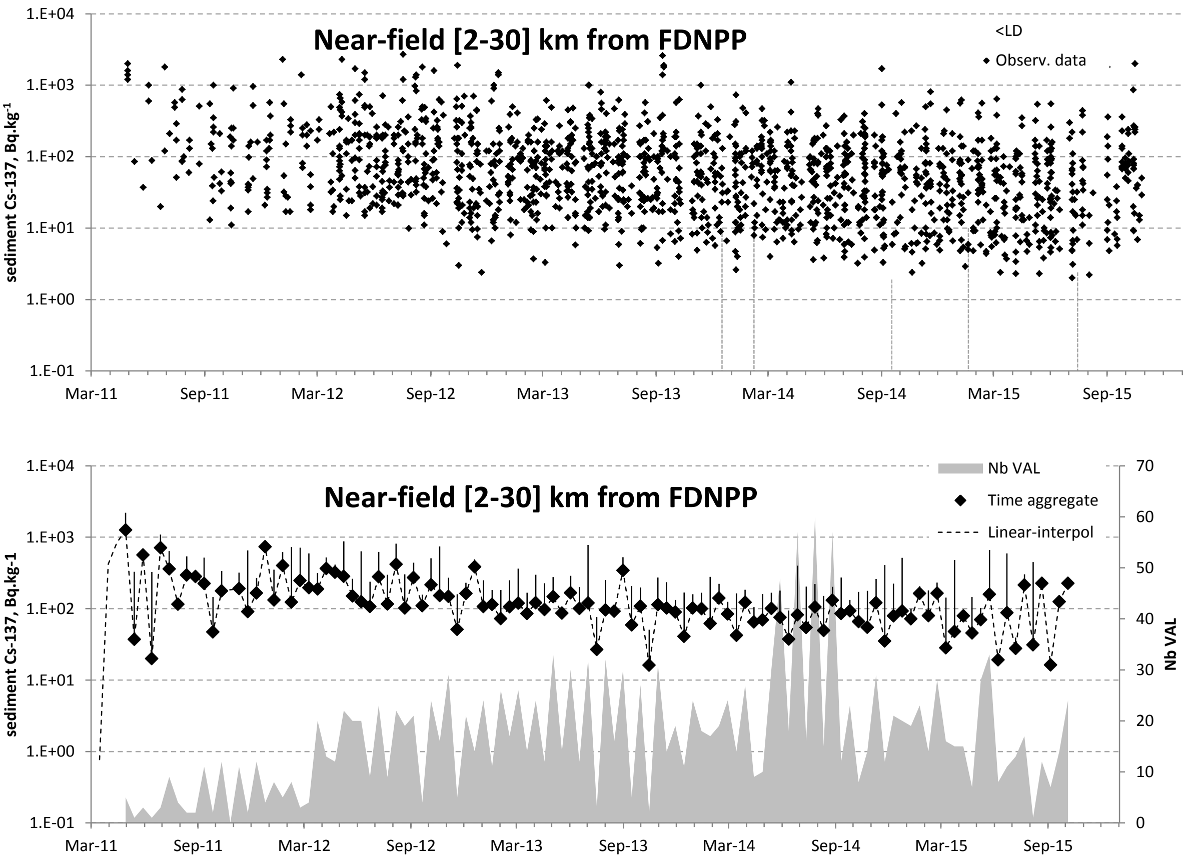

S3 Fig. Upper panel: Raw data of Cs-137 values (Left Y-axis in log scale) in sediment from the near-field ([2-30] km from FDNPP). Black dots: values above the LoD; vertical bars: censored values (<LoD). **Lower panel**: Filled diamonds and vertical bars: monthly averages + SD computed without the censored values because they were very few. Dark grey areas (Right Y-axis): the total number of values.
