# Supplementary material for: A dual pathways transfer model to account for changes in the radioactive caesium level in demersal and pelagic fish after the Fukushima Daï-ichi nuclear power plant accident
Source: PLoS One. 2017 Mar 1;12(3):e0172442. doi: 10.1371/journal.pone.0172442 (PMC5383001; doi:10.1371/journal.pone.0172442)
Supplement: S5 Fig — (PDF) [file pone.0172442.s006.pdf]

S5 Fig

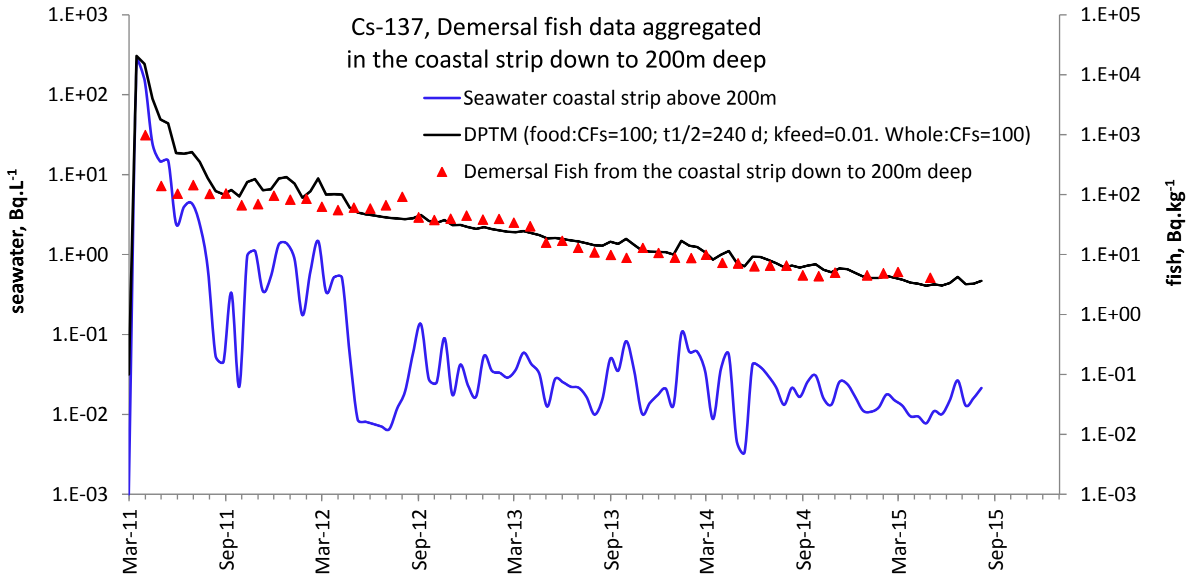

S5 Fig. Alternative near-field area delineated between latitude 35.7 N and 38.3 N and isobaths 200m. Blue solid line: seawater signal used as the input for the DPTM. Red close triangle: monthly averaged demersal fish data (all species aggregated) from the alternative near-field area. Solid black line: DPTM output computed with transfer parameters CFsfood and CFs = 100; $tb_{1/2}$ = 5 d; k_{feed} = 0.01 d^{-1}; $tb_{1/2}$food = 240 d.
