# Supplementary material for: A dual pathways transfer model to account for changes in the radioactive caesium level in demersal and pelagic fish after the Fukushima Daï-ichi nuclear power plant accident
Source: PLoS One. 2017 Mar 1;12(3):e0172442. doi: 10.1371/journal.pone.0172442 (PMC5383001; doi:10.1371/journal.pone.0172442)
Supplement: S6 Fig — (PDF) [file pone.0172442.s007.pdf]

S6 Fig

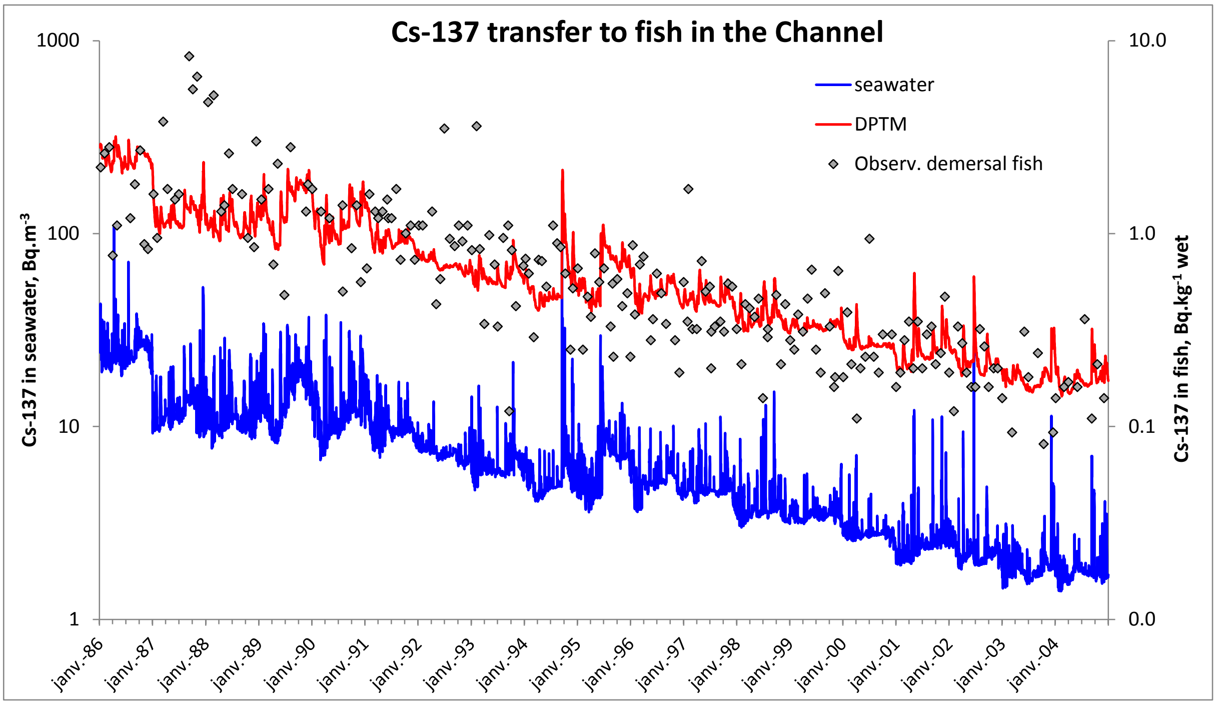

S6 Fig. Trial application of the DPTM in the English Channel Sea. Blue solid line: seawater signal computed 5 km North from the French Cotentin Peninsula with the hydrodynamic model (Bailly du Bois and Dumas, 2005) and used as the input for the DPTM. Grey filled diamonds: monthly individual demersal fish data (all species aggregated) caught in the near-field in the North of the Cotentin Peninsula. Red solid line: DPTM output computed with the same transfer parameters as for demersal fish in the near-field off Fukushima (CFsfood and CFs = 100; $tb_{1/2}$ = 5 d; k_{feed} = 0.01 d^{-1}; $tb_{1/2}$food = 240 d).
