# Supplementary material for: A dual pathways transfer model to account for changes in the radioactive caesium level in demersal and pelagic fish after the Fukushima Daï-ichi nuclear power plant accident
Source: PLoS One. 2017 Mar 1;12(3):e0172442. doi: 10.1371/journal.pone.0172442 (PMC5383001; doi:10.1371/journal.pone.0172442)
Supplement: S1 Table — (PDF) [file pone.0172442.s008.pdf]

S1 Table

model parameters f(transfer parameters)	transfer parameters f(model parameters)
$a_f = exp^{(-kf_{out}.T)}$	$CFs = \frac{k_{in} + k_{feed}.CFsfood}{k_{out}} = \frac{b + c.\frac{b_f}{(1-a_f)}}{(1-a)}$
$b_f = \frac{CFsfood}{1-a_f}$	$CFsfood = \frac{kf_{in}}{kf_{out}} = \frac{b_f}{1-a_f}$
$a = exp^{(-k_{out}.T)}$, with $k_{out} = k_b + k_p$	$k_{feed} = \frac{c.Ln\left(\frac{a_f}{a}\right)}{\left(1-\frac{a}{a_f}\right).T}$
$b = CFs.[1 - exp^{-k_{out}.T}] - c.CFsfood$	$tb_{1/2} = \frac{Ln(2)}{k_b}$, with $k_b = \frac{-Ln(a)}{T} - k_p$<br>($k_p$ = RN radioactive decay)
$c = \frac{k_{feed}}{(k_{out}-kf_{out})}.\left\{1 - exp^{[-(k_{out}-kf_{out}).T]}\right\}$<br>with $k_{out} \neq kf_{out}$ $(a \neq a_f)$	$tb_{1/2}food = \frac{Ln(2)}{kf_b}$<br>with $kf_b = \frac{-Ln(a_f)}{T} - k_p$

S1 Table: Equations relating the model parameters a_f, b_f, a, b, c and the transfer parameters CFs, CFsfood, k_{feed}, $tb_{1/2}$ and $tb_{1/2}$food.
