# Supplementary material for: A dual pathways transfer model to account for changes in the radioactive caesium level in demersal and pelagic fish after the Fukushima Daï-ichi nuclear power plant accident
Source: PLoS One. 2017 Mar 1;12(3):e0172442. doi: 10.1371/journal.pone.0172442 (PMC5383001; doi:10.1371/journal.pone.0172442)
Supplement: S1 Text — (PDF) [file pone.0172442.s009.pdf]

Mathematical background

The DPTM can be expressed as two differential equations

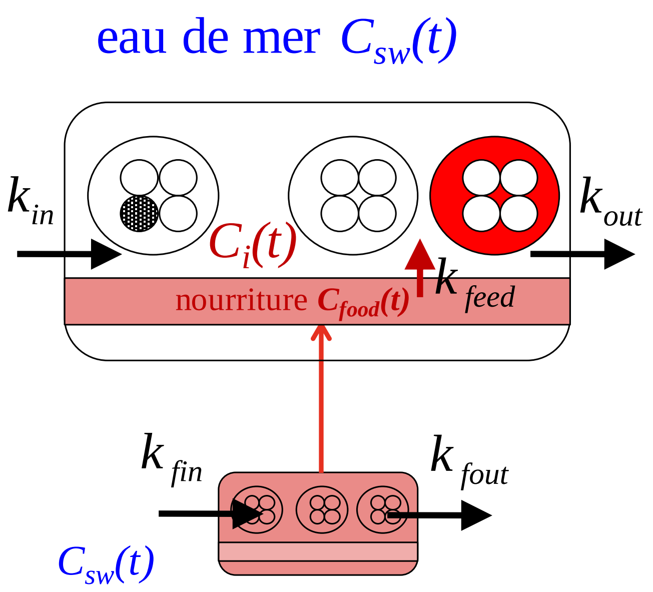

$$\frac{dC_{bio}(t)}{dt} = k_{in} \cdot C_{sw}(t) + k_{feed}.C_{food}(t) - k_{out} \cdot C_{bio}(t)$$

$$\frac{dC_{food}(t)}{dt} = k_{fin} \cdot C_{sw}(t) - k_{fout} \cdot C_{food}(t)$$

The usual variational method yields the following solution (**eq.1**)

$$C_{bio}(t) = \left(\frac{k_{in}}{k_{out}} + \frac{k_{feed}}{k_{out}} . \frac{k_{fin}}{k_{fout}}\right).C_{sw}.[1-\exp^{(-k_{out}.t)}] + \frac{k_{feed}.k_{fin}}{(k_{out}-k_{fout}).k_{fout}}.C_{sw}.[\exp^{(-k_{out}.t)} - \exp^{(-k_{fout}.t)}]$$

In the case where $k_{out} = k_{fout}$, the solution is

$$C_{bio}(t) = \left(\frac{k_{in}}{k_{out}} + \frac{k_{feed}.k_{fin}}{k_{out}^{2}}\right).C_{sw}.[1-\exp^{(-k_{out}.t)}] + \frac{k_{feed}.k_{fin}}{k_{out}}.C_{sw}.t.\exp^{(-k_{out}.t)}$$

We shall not develop this latter case any further because we are more interested in the cases where k_{out} # k_{fout}

Implementation of the differential equations solution in the DPTM

The DPTM writes as:

$$s_i = a.s_{i-1} + b.e_i + c.f_i$$
$$f_i = a_f.f_{i-1} + b_f.e_i$$

Note than the food compartment is a SPTM as described in Fievet and Plet 2003. So we already know that

$a_f = \exp^{[-kf_{out}.T]}$ and $b_f = CF_f.(1-a_f)$ with $CF_f = \frac{k_{fin}}{k_{fout}}$ (CF_f = CFsfood)

As it was previously done for the SPTM in Fiévet and Plet (2003), we can calculate s_i when e_i changes from 0 to 1 and check that we get eq.1

At time zero we can set $s_0 = 0;\ f_0 = 0$ and we can calculate s_i, f_i when i increments from 1 to n

$$f_1 = b_f;\ s_1 = b + c.b_f$$

$$f_2 = a_f.b_f + b_f;\ s_2 = a.(b + c.b_f) + b + c.(a_f.b_f + b_f);\ s_2 = a.b + b + (a + a_f + 1).c.b_f$$

and so on…

finally, $s_n = \sum_{i=0}^{n-1} a^i.b + \frac{c.b_f}{(1-a_f)}.[\sum_{i=0}^{n-1} a^i - \sum_{i=0}^{n-1} a^i.a_f^{n-i}]$

and since $\sum_{i=0}^{n-1} a^i = \frac{1-a^n}{1-a}$ we end up with **eq.2** $s_n = \frac{(1-a^n)}{(1-a)}.b + \frac{c.b_f}{(1-a_f)}.[\frac{(1-a^n)}{(1-a)} - \sum_{i=0}^{n-1} a^i.a_f^{n-i}]$

Let set $a = \exp^{[-k_{out}.T]}$ and since $a_f = \exp^{[-k_{fout}.T]}$ (see above), we can now write

$$\sum_{i=0}^{n-1} a^i.a_f^{n-i} = \sum_{i=0}^{n-1} (\exp^{[-k_{out}.T]})^i.(\exp^{[-k_{fout}.T]})^{n-i} = \sum_{i=0}^{n-1} \exp^{[-k_{out}.i.T - k_{fout}.(n-i).T]}$$

and by factorizing, we end up with $\sum_{i=0}^{n-1} a^i.a_f^{n-i} = \frac{\exp^{[-k_{fout}.n.T]} - \exp^{[-k_{out}.n.T]}}{(1-\exp^{[-(k_{out}-k_{fout}).T]})}$

eq.2 now writes $s_n = \frac{(1-a^n)}{(1-a)}.b + \frac{c.b_f}{(1-a_f)}.[\frac{(1-a^n)}{(1-a)} - \frac{\exp^{[-k_{fout}.n.T]} - \exp^{[-k_{out}.n.T]}}{(1-\exp^{[-(k_{out}-k_{fout}).T]})}]$

and finally,

$$s_n = \frac{b.(1-a_f) + c.b_f}{(1-a).(1-a_f)}.(1-a^n) + \frac{c.b_f}{(1-\exp^{[-(k_{out}-k_{fout}).T]}).(1-a_f)}.[\exp^{[-k_{out}.n.T]} - \exp^{[-k_{fout}.n.T]}]$$

At step n, t = n.T and C(t)=$s_{n.T}$, so we can express C(t) when C_{sw}=1 as

$$C(t) = \frac{b.(1-a_f) + c.b_f}{(1-a).(1-a_f)}.[1-\exp^{[-k_{out}.t]}] + \frac{c.b_f}{(1-\exp^{[-(k_{out}-k_{fout}).T]}).(1-a_f)}.[\exp^{[-k_{out}.t]} - \exp^{[-k_{fout}.t]}]$$

which is the same as equation eq.1 for C_{sw} = 1 if we set

$$\frac{k_{in}}{k_{out}} + \frac{k_{feed}.k_{fin}}{k_{fout}.k_{out}} = \frac{b.(1-a_f) + c.b_f}{(1-a).(1-a_f)} \text{ and } \frac{k_{feed}.k_{fin}}{(k_{out}-k_{fout}).k_{fout}} = \frac{c.b_f}{(1-\exp^{[-(k_{out}-k_{fout}).T]}).(1-a_f)}$$

We can finally express the equations between the DPTM model parameters *a_f, b_f, a, b* and *c*, and the transfer parameters CFsfood, $tb_{1/2}$food, k_{feed}, CFs and $tb_{1/2}$ in S1 Table ($T=t_i-t_{i-1}$ is the model computing time step).
